# Polycystic Ovary Syndrome: Important Underrecognised Cardiometabolic Risk Factor in Reproductive-Age Women

**DOI:** 10.1155/2015/786362

**Published:** 2015-06-01

**Authors:** Dinka Pavicic Baldani, Lana Skrgatic, Roya Ougouag

**Affiliations:** ^1^Division of Reproductive Endocrinology and Infertility, Department of Obstetrics and Gynecology, Clinical Hospital Centre, School of Medicine, University of Zagreb, Petrova 13, 10 000 Zagreb, Croatia; ^2^School of Medicine, Medical Studies in English, University of Zagreb, Šalata 3, 10 000 Zagreb, Croatia

## Abstract

Polycystic ovary syndrome (PCOS) is the most common endocrine disorder amongst women of reproductive age. Although PCOS is diagnosed exclusively based on reproductive criteria, it is also a metabolic disorder. Insulin resistance, impaired glucose tolerance, type 2 diabetes mellitus, obesity, and dyslipidemia are more common in women with PCOS than in age-comparable women without PCOS. Many of the metabolic abnormalities that manifest in PCOS are worsened by the concurrent incidence of obesity. However, some of these metabolic perturbations occur even in lean women with PCOS and therefore are rightfully recognized as intrinsic to PCOS. The intrinsic factors that produce these metabolic disturbances are reviewed in this paper. The consequences of obesity and the other metabolic aberrations are also discussed. The metabolic perturbations in PCOS patients lead to chronic low-grade inflammation and to cardiovascular impairments that heighten the risk of having cardiovascular disease. Even though many studies have shown an elevation in surrogate biomarkers of cardiovascular disease in PCOS women, it is still not clear to what extent and magnitude the elevation precipitates more frequent and earlier events.

## 1. Introduction

Polycystic ovary syndrome (PCOS) is the most common endocrine disorder amongst women of reproductive age. It is a heterogeneous disorder of uncertain etiology, but there is strong evidence that complex interactions between genetic, environmental, and behavioral factors contribute to causing this syndrome [1]. PCOS affects as many as 10% of reproductive-age women when using the NIH criteria for diagnosis, and up to 18% of reproductive-age women are diagnosed with PCOS as per the Rotterdam criteria [2]. Nevertheless, at least 70% of PCOS cases remain undiagnosed in primary care [3].

Although the diagnosis of PCOS is based exclusively on reproductive criteria (hyperandrogenism, oligo/anovulation, and/or PCO on ultrasound) [4], and management tends to focus primarily on treatment of infertility and hirsutism [5], PCOS is also a metabolic disorder. Women with PCOS have an increased risk of presenting with insulin resistance (IR) [6], impaired glucose tolerance (IGT) [7], type 2 diabetes mellitus (DM2) [7], obesity [6], and dyslipidemia [8]. In addition to presenting with these traditional risk factors for CVD, women with PCOS also show evidence of an increase of nontraditional, novel CVD risk factors, such as subclinical atherosclerosis [9] and an elevation in inflammatory markers [10]. As PCOS seems to be dominated by metabolic consequences, both as a consequence of the condition and as a vector for further complications, including DM2, CVD, and the exacerbation of the reproductive features of the syndrome (hirsutism and an/oligoovulation) [5], it is evident that research on the metabolic and cardiometabolic features of PCOS is needed. The present review is a contribution to this overall effort. The sections that follow discuss the cardiometabolic aspects of PCOS, their potential causes, their associated risks, and possible screening measures.

## 2. Methods

An extensive literature search was conducted to review publications from the late 1980s to the present. Online sources of medical databases included the US National Library of Medicine (NLM), the National Center for Biotechnology Information (NCBI) at the NLM, the Helios Group Central Medical Library, PubMed, and Medscape. The search was conducted with a combination of terms that included “PCOS,” “cardiometabolic,” “cardiovascular disease,” “metabolic syndrome,” “insulin resistance,” and “obesity” that were assumed to be relevant. Articles were also selected among references in the published papers found in the automated searches. Studies and review articles covering the focused areas were then selected.

## 3. Diagnostic Criteria of PCOS and Different PCOS Phenotypes

*Commonly Used Criteria*. Three different sets of criteria have been used for the diagnosis of PCOS for the past two decades: the National Institutes of Child Health and Human Development (NICHD) or what is known as the NIH criteria (developed in 1990), the Rotterdam criteria (adopted at a PCOS consensus meeting held in 2003), and the Androgen Excess (AE) and PCOS Society (AE-PCOS) criteria (proposed in 2006) [5]. These criteria are summarized in Table 1.

The different diagnostic criteria create several phenotypes of PCOS. Even before the Rotterdam criteria were adopted, it was evident that different subgroups of PCOS existed and it was even suggested that these subgroups differed metabolically [6]. One extensive review by Moran and Teede sought to compare the metabolic profiles among these different reproductive phenotypes [11]. For simplification, the phenotypes were divided into four diagnostic groups: phenotype A (NIH PCOS of biochemical/clinical hyperandrogenism and oligo/anovulation with PCO); phenotype B (NIH PCOS of biochemical/clinical hyperandrogenism and oligo/anovulation without PCO); phenotype C (non-NIH PCOS with biochemical/clinical hyperandrogenism and PCO but with normal ovulation); phenotype D (non-NIH PCOS with oligo/anovulation and PCO but without any biochemical/clinical hyperandrogenism) [11]. The diagnostic criteria of each of these four phenotypes are summarized in Table 2.

PCOS is also acknowledged as a metabolic disorder. Cardiometabolic features of PCOS are summarized as follows:

 visceral obesity,
 insulin resistance and hyperinsulinemia,
 risk of type II diabetes,
 disturbed secretion from adipocytes (adipokines, proinflammatory, and macrophage-derived factors),
 dyslipidemia,
 vascular endothelium dysfunction,
 prothrombotic state,
 atherosclerosis.

Most studies comparing the two subtypes of NIH PCOS, phenotypes A and B, report that women diagnosed under phenotype A present with few, if any, differences in metabolic profiles compared with women having phenotype B PCOS [11]. Similarly, most studies limiting comparison to only non-NIH subtypes, phenotypes C and D, also agree that women with phenotype C PCOS do not present with different metabolic risks compared to women with phenotype D PCOS [11]. However, most studies conclude that women with NIH PCOS (phenotypes A and B) present with more adverse metabolic profiles (including higher IR, increased prevalence of metabolic syndrome, and more adverse lipid profiles) than those with non-NIH PCOS (phenotypes C and D) [11].

**Figure 1:**
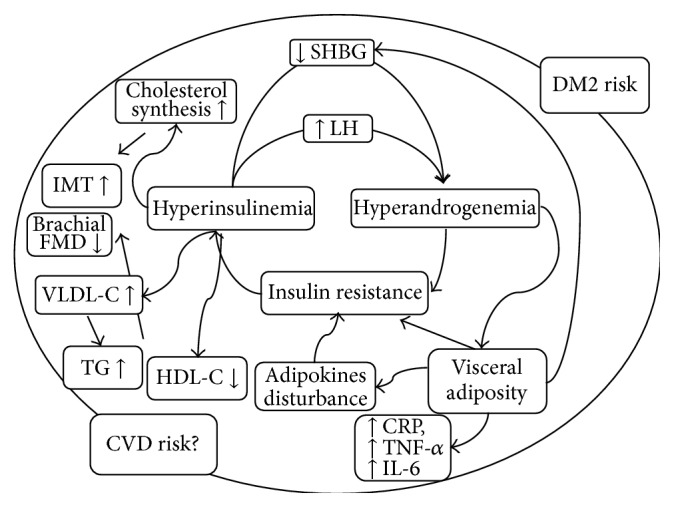
Pathophysiology of metabolic disturbances in PCOS. CVD, cardiovascular disease; TG, triglycerides; HDL-C, high-density lipoprotein cholesterol; VLDL-C, very-low-density lipoprotein cholesterol; CRP, C-reactive protein; TNF-*α*, tumor necrosis factor-alpha; IL-6, interleukin-6; FMD, flow-mediated dilatation; IMT, intima-media thickness; SHBG, sex hormone binding globulin; LH, luteinizing hormone; DM, diabetes mellitus.

Studies comparing women categorized under the NIH and non-NIH PCOS groups, but only after matching the subjects for BMI and WHR, found that the metabolic profiles (degree of IR, metabolic syndrome prevalence, and lipid profiles) are similar in the NIH and non-NIH PCOS women [11]. These results suggest that although NIH phenotypes present with more adverse metabolic profiles, the worse metabolic profile is not an inherent feature of NIH PCOS but is related to excess adiposity, particularly abdominal adiposity, which is more common in NIH PCOS groups [11]. The interactions between the different pathophysiologic factors of PCOS with metabolic syndrome are summarized in Figure 1.

The etiology of PCOS remains uncertain; however, due to a variety of predisposing genes that interact with environmental and lifestyle factors, PCOS is considered a complex genetic disorder [12]. A large number of population studies have focused on discovering genes that influence the development of PCOS using the candidate gene approach [13], but their findings have been mostly irreproducible. It is possible that a particular gene influences PCOS in one ethnic group but not in the others [12]. Various PCOS phenotypes presumably result from the interaction between multiple predisposing genomic variants, each exerting only minor effects, and strong environmental influences.

## 4. Cardiometabolic Aspects of PCOS

### 4.1. Obesity in PCOS

The prevalence of obesity among women with PCOS in the USA is 70 to 80%, almost twice as much as in the general US female population [6, 14]. Most studies report the prevalence of obesity in affected women outside the USA to be between 38 and 50% [6, 14]. Differences in diagnostic criteria, environmental factors, ethnicity, and lifestyle contribute to these variations [6, 15, 16]. Although less obesity is reported outside the USA, the prevalence of obesity among PCOS women outside the USA is still higher than that of women in the general population outside the USA [14]. As an example, 38% of Italian PCOS women are reported to be obese [14], but the reported prevalence of obesity in the general Italian female population is only 8% [17], highlighting a possible contribution of PCOS* per se*, in addition to lifestyle and other factors cited above, to the pathogenesis of obesity.

#### 4.1.1. Consequences of Obesity in PCOS

Obesity plays a role in the expression of metabolic features and other clinical manifestations of PCOS [6, 8, 18]. IR appears in normal weight PCOS women, but the frequency and magnitude increase with obesity [19, 20]. The magnitude of IR, quantified in one study by the insulin to glucose ratio, demonstrated a strong, positive, and linear correlation with body mass index (BMI) of the PCOS subjects [20]. Hepatic insulin resistance, characterized by reduced sensitivity to insulin's suppression of endogenous glucose production, only occurs in obese PCOS women [21]. Obese PCOS women also have a 10-fold increase in their risk of suffering from DM2 and a 7-fold increase of IGT compared with normal weight (BMI < 25 kg/m^2^) PCOS women [22]. An accelerated rate of conversion from IGT to DM2 is strongly dependent upon BMI [23].

Obesity in PCOS increases the patient's risk of developing cardiovascular disease. Among PCOS women, the prevalence of the metabolic syndrome, as in the general population, increases with increasing BMI and is highest in obese women with PCOS [17]. Studies report the prevalence of the metabolic syndrome in PCOS women in the USA to be 43–47%, twice more than in the age- and BMI-matched control population, suggesting that PCOS* per se*, possibly by promoting abdominal fat accumulation, increases the risk of acquiring the metabolic syndrome [17]. The effect of obesity, of causing chronic low-grade inflammation [24] with an elevation of inflammatory markers (such as CRP, TNF-*α*, and IL-6) that increase the risk of cardiovascular disease [25], is even more pronounced in PCOS. In PCOS and non-PCOS women, levels of TNF-*α* [26, 27], IL-6, and CRP [27] correlate directly with BMI, but overweight and obese PCOS women in some studies have presented with significantly higher levels of these inflammatory markers than their BMI-matched non-PCOS counterparts [10, 27].

#### 4.1.2. The Pathogenesis of Obesity in PCOS

The pathogenesis of obesity in PCOS is likely multifactorial [28].

One study reported that, in a group of lean PCOS women, serum glycerol levels, which reflect lipolytic activity, were lower than glycerol levels in BMI-matched control women [28]. The same authors also showed that the subcutaneous adipocytes of the lean PCOS women were larger in size and exhibited a lower response to catecholamine-stimulated lipolysis than the adipocytes of the BMI-comparable control women, suggesting that decreased lipolysis of subcutaneous adipocytes is an early alteration in PCOS, leading to enlarged subcutaneous fat cells and later to the development of obesity [28].

Another contributor to the high prevalence of obesity in PCOS might be mutations in the peroxisome proliferator-activated receptor-*γ* gene [29]. A higher frequency of C→T substitution in exon 6 of the peroxisome proliferator-activated receptor-*γ* gene has been reported in PCOS women than in BMI-matched controls [29]. This substitution enhances adipogenesis and increases the size of subcutaneous adipocytes, possibly leading to obesity [29].

Several studies also report that women with PCOS [30, 31] or with PCO morphology on ultrasound [32] have a higher prevalence of bulimic behavior, in part because of increased androgens [33], which increase appetite and inhibit impulse control [33]. After treatment with flutamide [34] or with antiandrogenic oral contraceptives [35], a reduction of binge eating and meal-related hunger, respectively, has been reported in bulimic women, supporting the idea that androgens may play a role in appetite dysregulation and in the development of obesity in PCOS [33]. Women with PCOS, in comparison to BMI-matched controls, also have reduced secretion of the gastrointestinal satiety peptide cholecystokinin [30] and have dysregulated secretion of the appetite-regulating gut hormone ghrelin [36, 37] that is independent of diet. These alterations may cause the reduction in satiety that has been reported by PCOS patients in comparison to BMI-matched control women [30, 37].

Additionally, it is widely believed that ghrelin's actions are mediated centrally by neuropeptide Y (NPY) and by the system of NPY fibers [36]. NPY acts centrally to increase appetite [38]. In a study on PCOS women, NPY levels were reported to be higher in obese and lean women with PCOS than in BMI-comparable control women [39].

#### 4.1.3. The Distribution of Adipose Tissue in PCOS

Subcutaneous abdominal fat and visceral fat both contribute to the development of IR [40]. Visceral fat furthermore creates a chronic low-grade inflammation [41] and is a surrogate marker for ectopic fat accumulations [42], which are responsible for many of the harmful effects of obesity [43, 44]. Many studies, based on anthropometric measures such as the waist-to-hip (WHR) ratio, suggest that there is a tendency in PCOS towards the accumulation of fat in these harmful areas such as the abdominal visceral region. It is known that fat distribution in the abdominal area is associated with more adverse metabolic profiles in PCOS [11, 45]. However, there is debate as to whether abdominal fat storage occurs more in PCOS than in weight-matched controls. Several studies quantifying abdominal subcutaneous and visceral adipose tissue by MRI [45, 46] and DEXA (which only quantifies total central abdominal fat) [47] found no difference in the volume of total abdominal fat or visceral fat between PCOS women and BMI-matched controls. On the contrary, Dolfing et al. demonstrated even less visceral fat accumulation in lean PCOS women compared to matched controls assessed by MRI [48]. However, in other studies, MRI [42] showed an increase in visceral and subcutaneous abdominal fat, and DEXA [49] showed an increase in the proportion of upper body fat in PCOS women compared to BMI-matched controls. The different results may depend on the small number of patients and controls and may also be related to the degree of obesity [42]. Studies in which the majority [41, 42, 50] or all [49] of the women were nonobese have reported a higher quantity of central abdominal fat in PCOS women than in BMI-matched controls. Results from a more highly powered study with over 200 patients and controls evaluated by DEXA support the suggestion that the disparities reported by different authors are related to the degree of obesity [41]. When the 220 subjects from the study were stratified according to BMI into obese and nonobese subgroups, there was no difference in the quantity of central abdominal fat between obese PCOS women and obese control women, but when limiting the comparison to the nonobese women, the quantity of central abdominal fat was higher in nonobese PCOS women compared to the quantity in nonobese control women [41]. These observations demonstrate that when obesity is present, most subjects display abdominal obesity, independently of being afflicted with PCOS or not [41]. However, when obesity is not present, PCOS patients stock a higher portion of their total adiposity in the abdominal region than do BMI-comparable controls [41]. Abdominal adiposity may therefore be a risk factor in nonobese PCOS women that confers on them adverse metabolic profiles compared to their BMI-matched non-PCOS counterparts [41]. Studies report that the quantity of central abdominal fat positively correlates with the degree of IR in nonobese PCOS women [41] and to the level of inflammatory markers [50]. DEXA accurately quantifies fat in different regions, is not operator dependent, and, unlike MRI, can be used on large populations [41]. DEXA may therefore be a useful screening tool for nonobese PCOS women susceptible to central abdominal fat accumulation and, hence, to the adverse metabolic complications associated with centripetal fat distribution [11].

### 4.2. Insulin Resistance and Hyperinsulinemia

Insulin resistance (IR) occurs in 30% of lean women with PCOS [8] and 95% of obese women with PCOS [51]. The presence of IR in Mediterranean populations of PCOS patients is somewhat less than that reported in other nations [52, 53]. South Asians in particular have high prevalence of insulin resistance and metabolic syndrome with central obesity in comparison with other PCOS-related ethnic groups of a similar BMI [54]. African American and Hispanic women are more obese and more prone to metabolic problems [55, 56]. The ethnic origin and cultural habits largely contribute to manifestations and risks of insulin resistance in PCOS [7]. Overall, 60–80% of women with PCOS present with elevated insulin levels [57–60].

Insulin is also a major regulator of many enzymes involved in lipoprotein metabolism [61, 62]. Resistance to insulin may contribute, in part, to the dyslipidemia observed in PCOS [16, 61]. A detailed description of the steps involved in lipoprotein metabolism is beyond the scope of this review. However, it has been proven that IR increases the hepatic secretion of VLDL and decreases the elimination of VLDL and of chylomicrons [19]. The persistence of VLDL and of chylomicrons in the circulation provides a major source for triglyceride (TG) production [62]. IR also leads to the more rapid clearance of apolipoprotein-a, a constituent of HDL-C, thus reducing the production and levels of HDL-C [62]. In several population-based studies, described in a paper by Miccoli et al., measures of IR correlated positively with levels of TG and VLDL-C and negatively with levels of HDL-C [62]. A study by Slowinska-Srzednicka and colleagues sought to elucidate the role of insulin resistance in the development of lipid abnormalities in women susceptible to PCOS [63]. In a group of women with polycystic ovaries, after adjustment for age, BMI, and sex hormones, regression analysis showed a strong positive association between fasting insulin levels and TG and VLDL-C levels and a negative association between fasting insulin levels and levels of the HDL constituent apolipoprotein-a [63].

The high prevalence of insulin resistance in PCOS also renders PCOS women 10 times more likely than controls to develop gestational diabetes and up to 5 times more likely to develop insulin-related complications such as spontaneous abortion [8].

#### 4.2.1. The Pathogenesis of Insulin Resistance in PCOS

The IR of PCOS is in part independent of obesity; it is primarily a result of intrinsic factors. A postbinding decrease in the phosphorylation of the tyrosine residues and an increase in the phosphorylation of the serine residues of the intracellular domain of the insulin receptor cause resistance to insulin's metabolic actions [6]. An elevation in serine phosphorylation not only decreases the responsiveness of the insulin receptor to its substrate, but also enhances the activity of P450C17, the key enzyme of adrenal and ovarian steroid synthesis [64]. The same defect in serine phosphorylation is therefore thought to cause both IR and hyperandrogenism in a subgroup of PCOS patients [6]. Other possible causes of insulin resistance in PCOS include increased serine phosphorylation of the adaptor protein IRS-1 [6]. Serine phosphorylation of the latter disrupts intracellular signaling necessary for the translocation of GLUT4 into the plasma membrane [6]. Reduced expression of GLUT4 has been demonstrated in the plasma membranes of adipocytes of both lean and obese PCOS patients [65]. Increased activation of ERK1/2 pathways in muscle cells of PCOS women may also be responsible for resistance to insulin's metabolic actions [6, 66]. Although ERK1/2 pathways are usually involved in insulin's mitogenic actions [6, 66], enhanced basal activation of ERK1/2 can also inhibit the IRS-1 pathways necessary for GLUT4 translocation to the plasma membrane [66]. Increased lipolysis in visceral fat cells may contribute to the hepatic insulin resistance observed in obese PCOS women [67]. Visceral fat cells of PCOS women demonstrate an enhanced lipolytic response to catecholamines [67]. Enhanced lipolysis of visceral fat raises fatty acid and glycerol delivery to the portal vein and liver, perturbing liver function, eventually leading to hepatic IR, as well as to hepatic inflammation and to interference with the production of SHBG [67].

### 4.3. Impaired Glucose Tolerance and Type 2 Diabetes

The American Diabetes Association has designated PCOS as a nonmodifiable risk factor for type 2 diabetes [68]. The prevalence of IGT and DM2 in women with PCOS, assessed in three large ethnically diverse US cross-sectional studies, was 23–35% for IGT and 4–10% for DM2, that is, twice the prevalence in age- and weight-matched healthy women without PCOS [6]. The prevalence of IGT and DM2 among PCOS women from other countries (Italy, Netherlands) was also found to be significantly higher than the prevalence in control women from the same region, although the overall proportion of European PCOS women having IGT or DM2 is nevertheless lower than that of US PCOS women affected by these conditions [6]. Authors suggest that different diagnostic criteria, diet, race, and ethnicity may account for the higher prevalence of IGT and DM2 in US PCOS women [6, 16]. When the PCOS women from European and US studies were stratified according to BMI and comparisons were limited to women in comparable BMI categories, the differences in the prevalence of IGT and DM2 between US and European PCOS women still persisted but decreased, highlighting the contribution of lifestyle to the disparities in IGT and DM2 observed between US and European women [6]. Additionally, a study of two PCOS populations in the USA, one urban ethnically diverse and one rural ethnically homogeneous, showed similar proportions of women with IGT and DM2 in each of the two populations, therefore demonstrating that PCOS may be a more important risk factor for IGT and DM2 than factors such as race and ethnicity [69]. These general tendencies towards a deterioration of glucose metabolism have been confirmed by a meta-analysis indicating a higher prevalence of IGT (odds ratio 2.54) and DM2 (odds ratio 4) in PCOS women than in BMI-matched controls [7].

Studies have also reported higher conversion rates from normal glucose tolerance (NGT) to IGT and from IGT to DM2 in PCOS women [70, 71]. IGT is an independent predictor of developing DM2 and CVD and of suffering mortality from CVD [71]. Early identification and treatment of IGT with lifestyle intervention and/or metformin have been shown to improve outcomes [72]. These observations have led experts at the most recent ESHRE/ASRM-sponsored PCOS consensus workshop to suggest the implementation of an annual screening of all PCOS women for IGT with the OGTT [3, 73], the most sensitive test for assessing IGT in PCOS [6].

Chronic hyperinsulinemia* per se* exacerbates IR, leading to a higher demand for insulin production and eventually to *β*-cell burnout, thereby accelerating the progression to IGT and DM2 [5]. Although women with PCOS have higher basal insulin secretion conditioned by chronic IR, they demonstrate *β*-cell secretory defects, manifested by reduced insulin secretory response to meals [74] and eventually an overall secretion of insulin that is inadequate for the degree of IR [75]. IR, *β*-cell secretory defects, and eventual *β*-cell burnout contribute to the development of IGT and DM2 in PCOS [74].

### 4.4. Adipokines

The increased incidence and severity of cardiovascular risk factors and of metabolic disturbances in PCOS may be in part related to the abnormal production and release of adipokines and inflammatory factors by adipose tissue [8]. Although traditionally regarded as a storage organ, emerging evidence also strongly suggests that adipose tissue is an endocrine organ [8], whose altered function may produce widespread cardiometabolic disturbances in PCOS. It is believed that dysregulated adipocyte function and obesity play a pathophysiological role in PCOS [5, 28].

#### 4.4.1. Leptin

Leptin, a protein secreted by adipocytes, suppresses an individual's appetite and promotes energy expenditure [76]. Serum leptin levels are elevated in obese patients, who are considered leptin resistant [76]. Hyperleptinemia seems to be a positive risk factor for cardiovascular disease [77–79]. Although some studies have found leptin levels to be elevated in PCOS women compared to controls [80, 81], the general consensus reported by the majority of published studies is that there is no difference in circulating leptin levels in PCOS subjects in comparison to BMI-matched controls [82–90]. The different results might be explained by differences in ethnicity, heterogeneity in criteria used to classify PCOS, and low number of PCOS subjects and controls [90, 91].

Most studies report that adiposity, quantified by BMI, is the main correlative component and determinant of leptin levels in PCOS women [82–90]. Leptin mRNA expression in adipocytes did not differ between PCOS women and BMI-matched controls [89], providing further evidence that obesity, rather than PCOS* per se*, affects leptin production and circulating levels. After adjustment for BMI, some authors report that leptin levels do correlate minimally with the free androgen index [82, 83, 86, 92] but nevertheless do not differ between visibly hirsute and nonhirsute women with PCOS [82]. Most studies addressing leptin and insulin report that, after adjustment for BMI, leptin levels in PCOS women do not correlate with the chronic insulin levels [82, 85, 88, 89, 92], while others report that leptin levels in PCOS women did correlate with measures of insulin resistance [81, 83]. However, in further support of findings that negate a significant correlation between insulin resistance and leptin levels, treatment of chronically hyperinsulinemic insulin resistant PCOS women with the thiazolidinediones troglitazone [85] or rosiglitazone [88] was shown to lower insulin levels but did not alter leptin levels in these patients.

#### 4.4.2. Adiponectin

Adiponectin, which is secreted exclusively by adipose tissue, exerts insulin sensitizing actions both indirectly [93] and directly by activating tyrosine phosphorylation of the skeletal muscle insulin receptor [94]. Adiponectin levels are reduced in insulin resistance states such as DM2 across all ethnic groups [95]. Low levels are also associated with a faster progression towards DM2 in at-risk individuals [95] and with higher risk of CHD in women [96]. Low levels are also possibly associated with high LH/FSH ratios and impaired ovulation because adiponectin in normal levels reduces secretion of LH through AMPK phosphorylation without affecting FSH secretion [97].

A meta-analysis has demonstrated that adiponectin levels are lower in PCOS women than in control women of comparable BMI [98]. A more recent meta-analysis has indicated that the T45G polymorphism in the adiponectin gene is associated with PCOS [99]. Although few studies exist focusing on high molecular weight adiponectin and the earlier meta-analysis did not specifically evaluate levels of high molecular weight (HMW) adiponectin [98], which is considered to be a more potent mediator of insulin sensitivity [100], it has been reported that levels of HMW adiponectin and the ratio of HMW adiponectin to total adiponectin are both lower in PCOS women than in age- and BMI-comparable controls [101].

#### 4.4.3. Visfatin

Visfatin is a cytokine secreted, among other cell types, by adipocytes [102]. It stimulates glucose uptake by cells, thus inducing insulin-mimetic effects [102]. A meta-analysis established that plasma visfatin levels are significantly increased in subjects with obesity, DM2, metabolic syndrome, and CVD [103]. Furthermore, in diabetics, serum visfatin levels increase with progressive *β*-cell deterioration [104]. Haider et al. demonstrated that insulin inhibited visfatin release from adipocytes in healthy subjects, suggesting that elevated visfatin levels may reflect insulin resistance [105].

Given visfatin's insulin-mimetic actions, some authors have suggested visfatin may be elevated to compensate for insulin resistance [106] and to prevent further resistance to insulin [107, 108]. However, elevated visfatin levels may produce harmful effects. Rising visfatin levels correlate with the degree of endothelial dysfunction, quantified by the decline in flow-mediated vasodilation and impaired renal clearance [109]. Visfatin activates nuclear transcription factor NF-*κ*B in vascular endothelial cells [110] and in lipid-laden macrophages of atherosclerotic lesions [111], culminating in the activation of metalloproteinase-2 [110] and metalloproteinase-9 [111, 112], leading to vascular inflammation and plaque destabilization, respectively.

In patients undergoing carotid endarterectomy or percutaneous coronary interventions, visfatin expression is higher in the atherosclerotic lesions of symptomatic patients than in the lesions of asymptomatic patients, further emphasizing the role of this adipokine in plaque destabilization and acute cardiovascular events [112]. Likewise, elevated visfatin levels in PCOS may also signal heightened cardiovascular risk in certain women with this syndrome, particularly in those with insulin resistance.

Given its associations with insulin resistance and vascular inflammation, several studies have been undertaken to elucidate the role of visfatin in PCOS. Higher levels of serum visfatin and visfatin mRNA in adipocytes have been reported in PCOS women compared to BMI-matched controls [106, 107, 113–115]. Serum visfatin levels were found to correlate with BMI [106, 113], insulin resistance [106, 107, 116], free androgen index [107], and LH levels [115].

It has been observed that metformin treatment for 3 months lowered visfatin levels [114]. However, the investigations demonstrated many interstudy variations in parameters such as BMI, IR, FAI, and LH that significantly correlated with visfatin levels in some studies but not in others. These variations may be attributed to the small number of participants, less than 30 PCOS women in all but one [107] of these investigations, and to interracial variations in the phenotypic expression of PCOS [106].

More recent studies have reported no differences in visfatin levels between PCOS women and controls [117, 118], therefore necessitating further inquiries with more participants to clarify the role of this adipokine in PCOS.

#### 4.4.4. Chemerin

Chemerin is a chemotactic protein secreted by adipocytes [119, 120] that is necessary for adipocyte differentiation [121]. It is able to attract macrophages, which express the chemerin receptor CMKLR1 (chemokine-like receptor 1) [122]. In view of its chemoattractant properties, this adipokine may be one factor underlying the link between obesity and chronic inflammation [122]. Chemerin also induces insulin resistance in peripheral tissues such as skeletal muscle by activation of ERK-1/2 and NF-*κ*B pathways, culminating in inhibited cellular glucose uptake [120]. Insulin stimulates chemerin secretion, promoting a vicious circle increasing insulin resistance [106]. This protein is thought to possibly present a link between obesity and diabetes [120]. Serum chemerin levels have been found to correlate with BMI [119, 120], WHR [119, 120], triglycerides [119], elevated blood pressure [119], and adipocyte volume [120]. The latter has been found to be higher in PCOS patients, even in lean PCOS patients when compared to BMI-matched controls [28]. Chemerin levels in PCOS are consequently of interest, as this may be one of the factors underlying the insulin resistance so common in PCOS. Chemerin is also implicated in inflammation, which may be responsible for vascular damage leading to CVD. Chemerin levels have been reported to be higher in obese PCOS women than in BMI- and WHR-matched controls [106, 123] as well as in lean PCOS women compared to BMI-matched controls [123]. Treatment of the PCOS patients with metformin for 6 months has lowered chemerin levels and improved insulin resistance, without changing BMI [106].

### 4.5. Proinflammatory and Macrophage-Derived Factors

This section presents a survey of macrophages and proinflammatory factors such as tumor necrosis factor-alpha (TNF-*α*), C-reactive protein (CRP), and interleukin-6 (IL-6).

#### 4.5.1. Macrophages

Adipose tissue inflammation mediated by activated tissue macrophages (ATMs) is a major pathway culminating in the development of obesity-related insulin resistance [124, 125]. CD11c is a marker specific to these ATMs that infiltrate adipose tissue in obese individuals and secrete cytokines such as TNF-*α* and IL-6, both of which are associated with insulin resistance. In contrast, the markers CD206, CD14, and CD163 are expressed by less inflammatory macrophages [125]. CD11c-expressing macrophages cluster around dead adipocytes, forming histologically defined crown-like structures (CLS) [125]. The density of CLS has been found to correlate with the degree of insulin resistance and obesity [125]. CD11c and CLS density is significantly higher in lean and obese PCOS women than in BMI-comparable non-PCOS controls [42]. The observation that CD11c macrophages and CLS occur more frequently in obese men than in obese women has led to the suggestion that the increase observed in lean and obese PCOS women is likely a result of hyperandrogenism [42]. CD11c adipose tissue infiltration and CLS may be an early change in lean hyperandrogenic PCOS women leading to the development of insulin resistance in this group [42] that is comparable to the insulin resistance in obese controls [19].

#### 4.5.2. Proinflammatory Factors

TNF-*α* plays a role in the pathogenesis of insulin resistance [126]. It inhibits tyrosine phosphorylation of the insulin receptor and of IRS-1 in muscle and fat cells [126] and has also been shown to downregulate the expression of the GLUT4 transporter necessary for cellular entry of glucose [127]. Serum levels of TNF-*α* are elevated in both obesity and DM2 [126]. Studies report that TNF-*α* levels in PCOS correlate with BMI [26, 27, 50, 128] and that circulating TNF-*α* levels are elevated in both nonobese [26, 129] and obese [27] PCOS women when compared with BMI-matched controls. However, in other studies, these differences in the levels of TNF-*α* between PCOS women and controls diminished after adjusting for BMI and abdominal adiposity [50, 128], thus questioning whether TNF-*α* elevations are related to PCOS or are a function of excess adiposity. A meta-analysis found no significant difference in TNF-*α* levels between PCOS subjects and BMI-matched controls [10]. However, the authors caution against overinterpretation of these results, as their study also revealed evidence of a publication bias favoring publication of studies that underestimate the differences in TNF-*α* levels between PCOS women and controls [10]. An important difference between PCOS and control subjects may lie in the TNF-*α* receptor [130]. Most of the metabolic effects of TNF-*α* are mediated through the TNF-*α* receptor 2 [130]. Although TNF-*α* receptor 2 levels were increased in obesity, no differences in levels of this receptor were observed between PCOS women and controls [130]. However, a methionine 196 arginine polymorphism in exon 6 of the gene encoding the TNF-*α* receptor 2 was reported to be significantly more frequent in women with PCOS than in controls, suggesting that TNF-*α* plays a role in the development of metabolic pathologies in PCOS and that this might be related to a structural change in the TNF-*α* receptor that confers a more responsive phenotype, rather than to circulating TNF-*α* levels or to the density of TNF-*α* receptors* per se* [130]. Authors agree that larger and more highly powered studies are needed to clarify the role of TNF-*α* in PCOS [10, 130].

CRP has been proven to be a strong independent predictor of cardiovascular events in healthy asymptomatic as well as symptomatic women in the general population [131, 132]. Obesity is associated with elevations in CRP [133]. A meta-analysis of 26 studies matching carefully for BMI revealed that CRP is elevated in PCOS independently of obesity [10]. This elevation of CRP in PCOS is more pronounced when obesity is present, further heightening the risk of cardiovascular events in this group of women [10]. However, the authors caution against overattributing increased cardiovascular risk to PCOS* per se* because, after adjusting for BMI, the elevation in CRP attributable to PCOS is relatively small [10].

Interleukin-6 (IL-6) is released by mononuclear leucocytes and adipose tissue [10], with levels being elevated in obesity [10]. It directly stimulates hepatic CRP synthesis [10]. Although IL-6 elevations have been reported in lean and obese PCOS women in relation to BMI-comparable controls [10], a recent meta-analysis proved no significant difference in circulating IL-6 levels between PCOS women and BMI-matched controls [10], suggesting that elevated IL-6 in PCOS is primarily related to obesity. However, a promoter region polymorphism (G/C) at position -174 of the gene encoding IL-6 has been found to be strongly associated with DM2 in the Caucasian population [134]. This same polymorphism has been reported to occur more frequently in PCOS patients [135, 136]. Furthermore, a certain microsatellite CA-repeat polymorphism in the locus encoding the *α*-subunit of the IL-6 receptor is associated with obesity, while the Arg148 allele in the region encoding the gp130 subunit of the IL-6 receptor gene is more common in normoandrogenic subjects than hyperandrogenic ones [137]. These observations suggest that genetically determined hypersignaling defects in the IL-6 receptor, rather than only circulating IL-6 levels, may be implicated in the pathogenesis of metabolic hyperandrogenic disorders such as PCOS [137]. However, relatively little is known about this field and authors agree that larger studies are needed [10, 137].

### 4.6. Dyslipidemia

Dyslipidemia is the most common metabolic abnormality in PCOS [4, 8], and polycystic ovary syndrome is the leading cause of dyslipidemia in reproductive-age women [138]. Observations of PCOS affected women and their relatives have shown that the probability of developing dyslipidemia is 1.8-fold larger in the PCOS individuals [8]. Overall, studies of PCOS patients report slightly decreased levels of cardioprotective HDL-C, with slightly elevated levels of TG, VLDL-C, and LDL-C [4, 8]. PCOS women display the lipid profile observed in insulin resistant states such as DM2 and characterized specifically by elevated TG and lowered HDL-C [8, 71]. The main determinant of heart disease risk is the total cholesterol (TC) to HDL-C ratio [51]. This ratio is also slightly elevated in PCOS patients [8].

Elevated LDL-C [71, 139–141] and VLDL-C [139] in PCOS are further elevated when excess adiposity is present, but, as confirmed by a recent meta-analysis, the higher levels occur in PCOS independently of obesity [142]. Elevated LDL-C levels are linked with hyperandrogenemia [139, 140, 143]. It is unclear whether hyperandrogenemia and elevated LDL-C have a causal relationship or whether these are closely related genetic traits inherited together [140].

Although LDL-C and VLDL-C are elevated in PCOS independently of obesity, obesity is thought to be the major determining factor for elevations in TG levels and for the reduction of HDL-C levels that are observed in PCOS [71, 140, 142, 144]. In a study comparing lipid profiles between PCOS probands, their sisters with and without PCO morphology on ultrasound, and controls, the elevated TG levels and reduced HDL-C levels in probands relative to the other groups disappeared after controlling for BMI [144], suggesting that BMI is the predominant determinant of TG and HDL-C levels in PCOS [144], both of which are strong independent risk factors for death from cardiovascular disease [145]. However, after adjustment for BMI, age, and centripetal obesity in another large study of PCOS women, HDL-C levels still remained significantly lower in PCOS women when compared to controls, though only slightly so [146]. This indicates that factors other than BMI and centripetal obesity are contributors to the lowering of HDL-C levels in PCOS women, although, of course, BMI is observably a significant determinant of lipid profiles [146].

In a study that did not control for diet, HDL-C levels were unexpectedly higher in obese PCOS women than in obese controls [71]. This inconsistency signals that, in addition to BMI [144], other factors such as age, ethnicity, genetic influences, and environment also modulate lipid profiles of women with PCOS [15, 71, 141, 147]. The importance of environmental (diet and activity level) and genetic contributions to dyslipidemia is evidenced by the fact that TG elevations in American PCOS women compared to Italian PCOS women persist even after controlling for BMI [15, 147].

Even PCOS women with normal lipid profiles may be at increased risk of cardiovascular events. This is because significantly higher levels of lipoprotein-a and a higher proportion of small, dense LDL have been found in PCOS patients compared to controls [148], although TC and total LDL-C levels did not differ between PCOS and control women [148]. This similarity in TC and total LDL-C levels makes the PCOS women appear to have normal lipid profiles. Such PCOS women are at higher risk of cardiovascular events, because certain lipoproteins, such as lipoprotein-a and small, dense LDL-C, are more atherogenic [148].

Another atherogenic shift in PCOS is the lipid-to-protein ratio of HDL-C particles. The lipid-to-protein ratio in an HDL-C particle reflects the capacity of the particle to remove cholesterol from tissues [149]. A reduction of this ratio signals a decreased or impaired capacity to remove cholesterol and prevent atherosclerosis [149].

In one study, the lipid-to-protein ratio in the HDL-C was found to be lower in obese PCOS women than in obese women without PCOS [149]. The latter finding signals a drop in the antiatherogenic properties of HDL-C of PCOS women [149].

Despite slight changes in lipid profiles in PCOS, most women with PCOS are young and have normal blood pressure and hence do not qualify for primary prevention of cardiovascular disease [8]. Nevertheless, performing at least one measurement of lipid profiles in PCOS in conjunction with an assessment for other cardiovascular risk factors such as smoking and family history of CVD is suggested [8].

### 4.7. Traditional and Novel Cardiovascular Risk Factors in PCOS

#### 4.7.1. Traditional Cardiovascular Disease Risks Factors in PCOS

Traditional risk factors for cardiovascular disease, such as IGT, DM2, dyslipidemia, obesity, and elevated blood pressure, are more prevalent in women with PCOS than in control women of similar age [51].

#### 4.7.2. Markers of Atherosclerosis

Calcification of the coronary arteries assessed by electron beam computed tomography correlates with the degree of atherosclerosis found on histopathological exam and was found to predict the incidence of cardiovascular events in asymptomatic women [150]. The prevalence and extent of coronary artery calcification (CAC) were found by several studies to be higher in both younger (aged 30 to 45 years) and older (aged over 40 years) women with PCOS than in controls, independently of age and BMI [151–153]. It has been suggested that the reported increase in CAC among PCOS women is related to the parameters that were different in PCOS women in relation to the control women in the studies: increased LDL-C [151, 153], lower HDL-C [153], and hyperinsulinemia [153]. Among the women with PCOS, BMI was a significant predictor of whether the women would have CAC [151–153], leading to the suggestion that obese women with PCOS should be targeted for aggressive treatment and prevention of cardiovascular disease [151, 152]. Talbott et al. also reported a higher prevalence and extent of aortic calcification (AC) in women with PCOS [153]. The investigators of the latter study found that total testosterone was an independent risk factor for greater AC [153]. In animal models, testosterone exacerbated atherosclerosis in female monkeys but conferred a protective effect in males [153]. Similarly, a large study reported that men with the highest total testosterone levels had a reduced risk of AC, but, conversely, women with elevated testosterone levels had the highest risk for CAC [153, 154], leading authors to suggest that the aorta in women may be more sensitive to the effects of endogenous testosterone [153, 154].

Increased intima-media wall thickness (IMT) is an early marker of atherosclerosis [155]. Increased carotid intima-media wall thickness (CIMT) is also a strong independent predictor of the occurrence of major cardiovascular events later in life [155]. Higher CIMT has been reported in both younger (age 20 to 35 years) [57, 156] and older (over 45 years) [153] patients with PCOS in comparison to controls of similar age and BMI. A recent meta-analysis indicated that women with PCOS had a 0.072 to 0.084 mm higher CIMT compared to controls [157]. Every 0.10 mm increase in CIMT has been estimated to increase the risk of a myocardial infarction (MI) by 15% and the risk of stroke by 18% [157]. The increase in CIMT in PCOS relative to controls of comparable age and BMI has been associated in different studies with higher levels of insulin [57], hyperandrogenism [156], Il-18 [157], LDL-C [157], and abdominal obesity [157], although the contribution of each of these factors to increased CIMT in PCOS has not been systematically evaluated [157]. However, CIMT increases with age in PCOS, as in the general population [157].

#### 4.7.3. Vascular Endothelial Dysfunction

Several studies have demonstrated decreased brachial artery flow-mediated dilation (FMD), a marker of endothelial function, in young normal weight, overweight, and obese women with PCOS compared to body mass matched controls [9, 17]. The decreased FMD was observed even in normal weight PCOS women who were also normotensive and had normal lipid profiles [9] and who therefore lacked many of the traditional cardiovascular risk factors [9]. It is considered that elevated androgen levels in the PCOS women relative to controls contribute to the observed decline in endothelial function [57].

An earlier study by Paradisi et al. further supports the role of elevated androgen levels in precipitating endothelial dysfunction [158]. When obese PCOS and control women of similar age, BMI, LDL-C, and TC levels received intrafemoral artery infusions of the endothelial-dependent vasodilator methacholine chloride (MCh), the leg blood flow was 50% less in the PCOS women compared to the controls, suggesting impaired nitric oxide (NO) production in the endothelial cells of PCOS women [158]. The degree of decrease in leg blood flow was strongly associated with free testosterone levels [158]. This, in conjunction with the observation that androgen deprivation in men has enhanced endothelial-dependent vasodilation [159], initially led to the suggestion that elevated androgen levels in PCOS women may be a major contributor to endothelial dysfunction and macrovascular disease [158].

Several molecules implicated in endothelial dysfunction have been linked to PCOS. A recent meta-analysis indicated that homocysteine, a mediator of endothelial injury, is in higher levels in PCOS women than in controls of similar age and BMI [160]. The same study also demonstrated that levels of asymmetric dimethylarginine (ADMA), a competitive inhibitor of endothelial NO synthase and an independent risk marker for cardiovascular morbidity and mortality [161], are higher in PCOS women than in age- and BMI-matched controls [160]. Several studies have found that, in comparison to age- and BMI-comparable control women, PCOS women also exhibit elevated levels of endothelin-1 [6, 57], a by-product of endothelial damage and a potent vasoconstrictor [57]. Plasminogen activator inhibitor-1, which inhibits fibrinolysis and in higher levels predisposes to accelerated development of atherosclerosis [162], has been shown to be elevated in normal weight young PCOS women relative to controls [163].

#### 4.7.4. Coagulation and Fibrinolytic Disturbances

Disturbances in circulating markers of coagulation and fibrinolysis may contribute to cardiovascular disease risk. Several studies observed dysregulation of the hemostatic system, particularly hypofibrinolysis, hypercoagulability [163, 164], and endothelial and platelet dysfunction, in women with PCOS [164, 165]. The potential mechanisms of coagulation disturbances remain to be elucidated. It has been observed that women with PCOS have high circulating concentrations of PAI-1 and fibrinogen, independent of age and BMI, correlated with low SHBG and high insulin levels [166]. Hyperinsulinemia impairs fibrinolysis by enhancing PAI-1 secretion and by inhibiting hepatic production of SHBG [167]. Our group recently observed strong negative linear association between serum SHBG and CRP levels, even following adjustment for BMI, WHR, TT, HOMA-IR, total cholesterol, LDL cholesterol, and triglyceride levels [168]. High CRP and sE-selectin were recently observed as the strongest explanatory factors of high fibrinogen levels in women with PCOS [166]. Strong positive correlation is recognized between hyperandrogenism and hypofibrinolysis in women with PCOS contributing to a prothrombotic state [163].

#### 4.7.5. Cardiac Dysfunction

Studies report that, compared to age- and BMI-matched controls, young PCOS women have increased left ventricular mass index (LVMi) [61], a predictor of CVD morbidity and mortality [8], and decreased diastolic filling [61, 169]. Both of these abnormalities occur independently of excess weight, presenting in lean as well as overweight and obese PCOS patients. Additionally, decreased left ventricular ejection fraction has been reported in young overweight and obese women with PCOS compared to controls [61].

#### 4.7.6. The Risk of Cardiovascular Events in PCOS

Even though many studies have shown an elevation in surrogate biomarkers of cardiovascular disease in PCOS women, the question remains as to what extent this translates into more frequent or earlier events. Only a few prospective epidemiological studies have addressed this question.

A 49-year follow-up study of 786 women diagnosed with PCOS based only on ovarian wedge section found that the risk of fatal cardiovascular events was not different between PCOS women and controls [170]. Similarly, a prospective 21-year follow-up study of 31 women with histologically verified Stein-Leventhal syndrome found that although the CVD risk factors hypertension and hypertriglyceridemia were still more prevalent among the PCOS women in postmenopausal age, these women did not have an increased risk of suffering MI, stroke, or death caused by CVD compared to non-PCOS women [171]. A retrospective study of 319 women diagnosed with PCOS based on more stringent criteria (an/oligoovulation and hyperandrogenism) reported that there was no difference in cardiovascular mortality risks between PCOS women and age-matched controls [172], although the PCOS women demonstrated a higher risk of nonfatal cerebrovascular events, even after adjustment for BMI [172].

Another investigation followed 82,439 women aged 20–35 for 14 years [173]. Compared with women reporting a history of regular menses, women reporting a history of very irregular menses had a significantly higher risk of nonfatal and fatal cardiovascular disease, even after adjustment for BMI, age, menopausal status, and smoking [173]. Although these women were not diagnosed with PCOS, it is estimated that 80–90% of women reporting menstrual irregularity have PCOS [8]. Furthermore, a recent meta-analysis indicated a 2-fold increased risk of coronary heart disease (CHD) and stroke for patients with PCOS relative to women without PCOS [174]. The meta-analysis found that there is a 55% increase in the risk for CHD and stroke in PCOS women using only studies that adjusted for BMI, showing that BMI is not the sole cause of increased risk of cardiovascular events in women with PCOS [174].

The risk of cardiovascular events also appears to be higher in postmenopausal women with a history of PCOS than those without. The Women's Ischemia Syndrome Evaluation study reported that the cumulative 5-year event-free survival for women without a history of PCOS is 88.4% and only 78.9% for those with a premenopausal history of PCOS [175].

A retrospective study found similar results. The study evaluated the incidence of CV events (MI, angina, heart failure, stroke, and CV death) in a cohort of 2300 PCOS women between 1988 and 2009 [176]. Overall, CV events were not any more prevalent in the cohort than in the local female population [176]. However, when the cohort was stratified by age and comparisons were limited to age-similar groups in the local female population, PCOS showed an association with CV events within each age group [176]. The age-specific prevalence of CV events was significantly higher in PCOS patients over 45 compared with the local female population, with odds ratio as high as 12.88 in women over 65 with a premenopausal history of PCOS [176]. Factors in the cohort associated with an increased risk of CV events were age, hypertension, obesity, smoking, and having DM2 [176].

## 5. Conclusions

This review has provided a survey of many intrinsic causative factors that underlie IR, obesity, and the other metabolic perturbations associated with PCOS. Future investigations may elucidate which of these intrinsic causative factors are present in the different phenotypic subgroups of PCOS women. Further studies may also delineate which of these intrinsic factors are more common in certain geographic regions and associated ethnicities. Finally, and more importantly, through future investigations we may gain an understanding of which of these causative factors are associated with the most severe consequences. This may help foster a better understanding of the pathophysiology underlying PCOS in different subgroups and populations. Such knowledge could then be leveraged to devise the most optimal screening and effective management for women from different subgroups and ethnicities.

Experts have advocated the annual screening of all PCOS women for IGT with the OGTT and more frequent screenings for those with other DM2 risk factors, such as a family history of DM2. Some investigators have also suggested screening lean women with DEXA for excess abdominal fat accumulation, as lean PCOS women with a higher proportion of abdominal fat relative to BMI-comparable control women are more susceptible to developing insulin resistance and may therefore benefit from more aggressive prevention.

Central abdominal fat accumulation has been associated with insulin resistance, chronic inflammation, and harmful ectopic fat accumulations. Studies evaluating abdominal fat accumulation in PCOS women relative to BMI-comparable controls have reported contradictory results. This highlights the need for more studies that would quantify ectopic, visceral, and subcutaneous abdominal fat by CT or MRI, in order to provide definitive answers about the relationship between PCOS and visceral fat.

Uncertainty also remains in PCOS regarding the incidence of cardiovascular disease later in life, despite the indisputable presence of multiple CV risk factors earlier in the lifespan. Therefore, prospective observational trials are urgently needed that follow patients diagnosed with PCOS based on strict inclusion criteria and that track these women from a young age until after menopause.

The most urgent problem with the current management of PCOS is that many doctors focus on the short-term cosmetic and reproductive consequences, while metabolic and psychological risks are often not considered. Indeed, current knowledge clearly indicates that metabolic complications are present in both lean and obese women with PCOS. Early screening and close follow-up are therefore encouraged in both groups of patients.

Also, the observed trends of insulin resistance incidence and early cardiovascular event occurrences within families point to the conclusion that the family members of PCOS women should probably be screened for metabolic disturbances.

## Figures and Tables

**Table 1:** PCOS diagnostic criteria, adapted from Teede et al. 2010 [5].

NIH 1990	Rotterdam 2003	AE-PCOS Society 2006
Both of the following^*^: (i) chronic anovulation, documented by oligo-or amenorrhea (ii) clinical and/or biochemical signs of hyperandrogenism (with exclusion of other etiologies, e.g., congenital adrenal hyperplasia) with or without PCO on ultrasound	At least two of the following^*^: (i) chronic anovulation, documented by oligo-or amenorrhea (ii) clinical and/or biochemical signs of hyperandrogenism (iii) polycystic ovaries (by ultrasound)	(i) Clinical and/or biochemical signs of hyperandrogenism and at least one of the following^*^: (i) ovarian dysfunction (oligo/anovulation and/or polycystic ovarian morphology)

^*^After exclusion of the diseases that produce a similar clinical picture.

**Table 2:** Diagnostic phenotypes of PCOS, adapted from Moran and Teede (2009) [11].

Phenotype A	NIH PCOS: hyperandrogenism and oligo/anovulation with PCO

Phenotype B	NIH PCOS: hyperandrogenism and oligo/anovulation without PCO

Phenotype C	Non-NIH PCOS: hyperandrogenism with PCO but with normal ovulation

Phenotype D	Non-NIH PCOS: no hyperandrogenism but with oligo/anovulation and with PCO
